# Cell-cycle-phase progression analysis identifies unique phenotypes of major prognostic and predictive significance in breast cancer

**DOI:** 10.1038/sj.bjc.6604924

**Published:** 2009-02-24

**Authors:** M Loddo, S R Kingsbury, M Rashid, I Proctor, C Holt, J Young, S El-Sheikh, M Falzon, K L Eward, T Prevost, R Sainsbury, K Stoeber, G H Williams

**Affiliations:** 1 Department of Pathology and Cancer Institute, The Paul O'Gorman Building, University College London, Gower Street, London WC1E 6BT, UK; 2 Wolfson Institute for Biomedical Research, University College London, The Cruciform Building, Gower Street, London WC1E 6BT, UK; 3 Department of Life Sciences, Faculty of Science and Technology, Anglia Ruskin University, East Road, Cambridge CB1 1PT, UK; 4 Department of Public Health and Primary Care, Centre for Applied Medical Statistics, University of Cambridge, Institute of Public Health, Forvie Site, Robinson Way, Cambridge CB2 0SR, UK; 5 Department of Breast Surgery, Princess Ann Hospital, Southampton SO16 5YA, UK

**Keywords:** Aurora A, DNA replication licensing, aneuploidy, prognosis, breast cancer, predictive testing

## Abstract

Multiparameter analysis of core regulatory proteins involved in G1–S and G2–M cell-cycle transitions provides a powerful biomarker readout for assessment of the cell-cycle state. We have applied this algorithm to breast cancer to investigate how the cell cycle impacts on disease progression. Protein expression profiles of key constituents of the DNA replication licensing pathway (Mcm2, geminin) and mitotic machinery (Plk1, Aurora A and the Aurora substrate histone H3S10ph) were generated for a cohort of 182 patients and linked to clinicopathological parameters. Arrested differentiation and genomic instability were associated with an increased engagement of cells into the cell division cycle (*P*<0.0001). Three unique cell-cycle phenotypes were identified: (1) well-differentiated tumours composed predominantly of Mcm2-negative cells, indicative of an out-of-cycle state (18% of cases); (2) high Mcm2-expressing tumours but with low geminin, Aurora A, Plk1 and H3S10ph levels (S–G2–M progression markers), indicative of a G1-delayed/arrested state (24% cases); and (3) high Mcm2-expressing tumours and also expressing high levels of the S–G2–M progression markers, indicative of accelerated cell-cycle progression (58% of cases). The active cell-cycle progression phenotype had a higher risk of relapse when compared with out-of-cycle and G1-delayed/arrested phenotypes (HR=3.90 (1.81–8.40, *P*<0.001)), and was associated with Her-2 and triple negative subtypes (*P*<0.001). It is of note that high-grade tumours with the G1-delayed/arrested phenotype showed an identical low risk of relapse compared with well-differentiated out-of-cycle tumours (HR=1.00 (0.22–4.46), *P*=0.99). Our biomarker algorithm provides novel insights into the cell-cycle state of dynamic tumour cell populations *in vivo*. This information is of major prognostic significance and may impact on individualised therapeutic decisions. Patients with an accelerated phenotype are more likely to derive benefit from S- and M-phase-directed chemotherapeutic agents.

Cancer is a heterogeneous and complex group of diseases caused by the accumulation of genetic lesions, which increase the activity of regulatory genes that drive cell proliferation and decrease the activity of proteins that normally inhibit it. Activation of dominant stimulatory oncogenes or inactivation of recessive tumour suppressor genes can affect all levels of growth-signalling pathways, including mitogens, mitogen growth factor receptors PI3kinase–Akt, Ras, Raf and ABL, upstream of molecules such as p16INK4A, Cyclin D, Myc, Cyclin E, p53, and downstream of pRB. Global gene expression profiling is ideally suited for analysis of the complex multifactorial, interactive and stepwise alterations in gene expressions that characterise tumorigenesis (Perou *et al*, 1999; Ross *et al*, 2000). The analysis of complex and redundant pathways that control proliferation, differentiation, apoptosis and DNA damage response by global genome-wide analysis is an intensive area of investigation aimed at identifying unique molecular signatures and biomarkers of prognostic and predictive significance in cancer (Van ‘t Veer *et al*, 2002; Paik *et al*, 2004). However, the actual performance of prediction rules using gene expression profiling has not turned out to be as informative as initially expected for many tumour types, and the list of genes identified can be highly unstable (Michiels *et al*, 2005; Dunkler *et al*, 2007). An alternative approach is to focus directly on the cell-cycle machinery, which acts as an integration point for information transduced through upstream pathways (Stoeber *et al*, 2001; Gonzalez *et al*, 2005; Williams and Stoeber, 2007).

The cell cycle represents a highly regulated series of events that leads to eukaryotic cell reproduction. Early in the cycle, the DNA is replicated and the chromosomes are duplicated during transit through S phase. This process begins at specific DNA sites called replication origins. At these sites, the DNA replication licensing machinery opens the DNA double helix, exposing it to the enzymes that carry out DNA synthesis (Machida *et al*, 2005). S phase is followed by chromosomal segregation, nuclear division and cell division, which is collectively called M phase. Most cell cycles contain additional gap phases between S and M phases, which provide additional time for growth and also serve as important regulatory transitions, through which progression to the next cell-cycle stage can be controlled by intracellular and extracellular signals (Nigg, 2001; Sherr and Roberts, 2004) (Figure 1A). G1 is a particularly important regulatory period, because it is here that most cells become committed to either continued division or exit from the cell cycle (Zetterberg and Larsson, 1985; Planas-Silva and Weinberg, 1997). In the presence of unfavourable conditions or inhibitory signals, cells can normally withdraw from the cell cycle into quiescent (G0), terminally differentiated or senescent out-of-cycle states, a characteristic feature of most of the functionally differentiated cells of the human body (Stoeber *et al*, 2001; Blow and Hodgson, 2002). Cancers, in contrast, are characterised by uncontrolled cell growth and, therefore, contain a high proportion of cycling cells. It is for this reason that many of the chemotherapeutic agents and newly emerging small-molecule inhibitors are cell-cycle-phase-specific.

The DNA replication licensing machinery represents a complex of initiator proteins, which bind and open the DNA at origins establishing replication forks. During late mitosis and early G1 phases, there is a sequential assembly of the replication licensing factors, ORC, Cdc6, Cdt1 and Mcm2–7, at replication origins to form pre-replicative complexes, rendering origins ‘licensed’ for DNA synthesis during S phase. At the G1–S transition, cyclin-dependent kinases and the ASK-dependent Cdc7 kinase trigger a conformational change in the pre-replicative complex, referred to as ‘origin firing’, resulting in the recruitment of Cdc45, Mcm10 and additional initiator proteins, which collectively promote origin unwinding and the recruitment of DNA polymerases (Machida *et al*, 2005). Expression of the licensing repressor, geminin, during S–G2–M phases prevents inappropriate re-initiation events through its interaction with Cdt1, resulting in a block to Mcm2–7 loading to chromatin (Hook *et al*, 2007). We and others have shown that the Mcm2–7 replication licensing factors, constituents of the heterohexameric DNA replicative helicase, are expressed throughout all cell-cycle phases (G1–S–G2–M), but are tightly downregulated during exit into out-of-cycle states (Stoeber *et al*, 2001; Barkley *et al*, 2007; Williams and Stoeber, 2007). The repression of origin licensing contributes to replication arrest and loss of proliferative capacity, as cells exit the mitotic cycle into the out-of-cycle state (Blow and Hodgson, 2002). This allows a functional distinction between the proliferative state and the non-proliferative out-of-cycle state, depending on whether origins are licensed (Blow and Hodgson, 2002). Detection of Mcm2–7 is therefore a powerful way of assessing the proliferative potential of the cell. We and others have shown that these unique biomarkers can clearly distinguish between cycling cells and the out-of-cycle state in a range of tissue types, including premalignant and malignant disorders (Freeman *et al*, 1999; Gonzalez *et al*, 2005; Williams and Stoeber, 2007). As neoplastic cells are characterised by uncontrolled proliferation, Mcm expression is currently being exploited as a cancer diagnostic marker in a broad range of tumour types (Gonzalez *et al*, 2005; Tachibana *et al*, 2005; Williams and Stoeber, 2007).

The rigorous control of mitotic events (M phase) is essential for successful completion of sister-chromatid segregation and cell division. Although cyclin-dependent kinases are the master regulators of mitotic entry, they do not act alone. Polo-like kinase 1 (Plk1), Aurora A and Aurora B are three additional protein kinases that control a subset of critical mitotic events, including centrosome maturation and separation, chromosome orientation and segregation (Nigg, 2001). These mitotic kinases are currently the focus of major clinical interest, as small-molecule inhibitors targeting these enzymes have potent tumour-killing effects (Keen and Taylor, 2004; Plyte and Musacchio, 2007). We have shown earlier in human cells and tissues that, similar to geminin, endogenous levels of Aurora A/B and Plk1 are tightly regulated in a cell-cycle-dependent manner, which is undetectable in G1 phase, accumulates during S phase and reaches a peak in G2/M phase, followed by a rapid degradation at the end of mitosis (Kulkarni *et al*, 2007). Histone H3 is a substrate for the Aurora kinases and is phosphorylated on Serine 10 only in mitosis (Crosio *et al*, 2002). Therefore, geminin, Aurora A/B and Plk1 represent biomarkers of the S–G2–M progression, whereas phosphohistone (H3S10ph) represents a biomarker of the M-phase transition (Gurley *et al*, 1978; Paulson and Taylor, 1982; Wei *et al*, 1999; Crosio *et al*, 2002; Wohlschlegel *et al*, 2002; Kulkarni *et al*, 2007; Williams and Stoeber, 2007) (Figure 1B).

Multiparameter analysis of Mcm2–7, geminin, Aurora A, Plk1 and H3S10ph, core regulators of the G1–S and G2–M transitions, thus allows a detailed analysis of the kinetics of complex dynamic tumour cell populations. This biomarker set not only allows the out-of-cycle state to be distinguished from G1 but also provides an assessment of cell-cycle progression and cell-cycle-phase distribution (Williams and Stoeber, 2007) (Figure 1B). These proteins are readily detectable immohistochemically in surgical biopsy tumour samples and can, therefore, be used to determine the cell-cycle dynamics of individual patient samples. Interestingly, the proliferation signature, which includes cell-cycle regulatory proteins, has emerged as one of the most prominent prognostic gene-expression patterns in genome-wide analysis studies (Whitfield *et al*, 2006). Here we therefore set out to test the hypothesis whether a tumour's cell-cycle phenotype might also provide information regarding *in vivo* behaviour and disease progression, and whether the cell-cycle-phase distribution analysis might also provide a guide for selection of patients most likely to benefit from cell-cycle-phase-specific chemotherapeutic agents (Figure 1C).

Breast cancer was selected as the tumour model system of choice to test this novel cell-cycle algorithm because current prognostic and predictive tools for this common malignancy have limited discriminant utility, and adjuvant chemotherapy for this particular tumour type utilises cell-cycle-phase-specific agents (Michiels *et al*, 2005, 2007; Dunkler *et al*, 2007). The best prognostic tool described to date for identifying which patients are most likely to benefit from adjuvant chemotherapy is the Nottingham Prognostic Index (NPI), which includes morphological correlates of biological features of aggressive disease (Elston and Ellis, 1991). This includes tumour size, lymph node spread and grade. The latter is a combined index of differentiation status, nuclear pleomorphism reflecting genomic instability and number of cells in M phase (Elston and Ellis, 1991; Rampaul *et al*, 2001). Patients can be divided into good (NPI score <3.4; 15-year survival rate 80%), moderate (NPI score: 3.4–5.4; 15-year survival rate: 42%) and poor (NPI score: >5.4; 15-year survival rate: 13%) prognostic groups, on which therapeutic decisions can be based (Elston and Ellis, 1991). However, up to one-third of women with negative axillary lymph nodes will suffer recurrence, whereas approximately one-third of node-positive patients not receiving adjuvant therapy remain recurrence-free after 10 years (EBCTCG, 1998a, 1998b). We have conducted an analysis of the G1–S and G2–M regulators to determine the relationship between cell-cycle state, tumour differentiation status and the acquisition of genomic instability in breast cancer, and how this cell-cycle phenotype might impact on *in vivo* behaviour. We have also investigated whether the cell-cycle phenotype provides additional prognostic information independent of the gold standard NPI, and how this might influence the selection of patients for neoadjuvant or adjuvant cell-cycle-phase-specific therapy (Figure 1C).

## Materials and methods

### Study cohort

A total of 182 patients diagnosed with invasive breast cancer between 1999 and 2004 were identified from the Breast Cancer Database held in the Department of Surgery at University College London (UCL) Hospitals (London, UK). Patients were selected on the basis of available histological material. Histological specimen had been reviewed by a qualified breast pathologist at diagnosis and assessed for histological subtype and nuclear grade according to the World Health Organization (WHO) criteria. All patients studied underwent a regular postoperative clinical assessment and contributed to the cross-sectional analyses. Ten patients were lost to follow-up, but five were known to have had recurrent cancer, of whom two died. A total of 167 patients contributed to the prospective analyses of survival and relapse, of whom 24 (14%) died from cancer within the study period, 12 died from other unrelated causes and 131 were still alive at last follow-up. There were 40 (24%) relapse events comprising relapses and deaths from cancer. The median follow-up period was 47 months (range: 1–92). The mean time to relapse was 26 months (s.d.=15, range: 2–55). The mean follow-up time among those who had not yet relapsed was 52 months (s.d.=20, range: 2–92). The mean survival time among those who died was 21 months (s.d.=12, range: 4–44). The mean follow-up time among those still alive was 50 months (s.d.=21, range: 1–92).

Formalin-fixed, paraffin-embedded breast tissues from these patients were retrieved from the archives of the Department of Pathology (UCL Hospitals, London, UK), and included all three histological grades (1–3) calculated according to the Nottingham modification of the Bloom and Richardson method (Elston and Ellis, 1991). Histological reports and specimens were available for all cases. These included 142 invasive ductal carcinomas, 26 lobular, 4 mucinous, 1 micropapillary and 9 of mixed type. Breast cancers were also subdivided on the basis of their hormone receptor status, Her-2 expression and basal cytokeratin (CK 5) expression. Using this immunohistochemistry-based approach, cancers were subdivided into three groups: (1) ER/PR+, Her-2+/− (*n*=145); (2) ER/PR−, Her-2+ (*n*=11); and (3) ER/PR−, Her-2− (*n*=26). These subgroups are clinically relevant and also approximate to the ‘luminal’, ‘Her-2’ and ‘triple negative/basal-like’ breast cancer subtypes, earlier defined by microarray-based gene expression profiling (Sorlie *et al*, 2001). Parameters recorded include date of birth, histological grade, tumour size, tumour type, lymph node status, lymphovascular invasion (LVI), date of diagnosis, date of relapse, date of last follow-up, and date and cause of death. The NPI was calculated according to the following formula: NPI score=0.2 × tumour size+tumour grade+nodal status (Rampaul *et al*, 2001). Randomly selected cases of normal breast tissue from 21 premenopausal women who had undergone reduction mammoplasty were additionally included in the study. Local research ethics committee approval for the study was obtained from the joint UCL/UCLH Committees on the Ethics of Human Research.

### Antibodies

A rabbit polyclonal antibody against human geminin was generated as described (Wharton *et al*, 2004). Ki67 monoclonal antibody (MAb) (clone MIB-1) was obtained from DAKO (Glostrup, Denmark), Mcm2 MAb (clone 46) from BD Transduction Laboratories (Lexington, KY, USA), oestrogen receptor-*α* (ER) MAb (clone 1D5) and progesterone receptor (PR) MAb (clone PgR 636) from DAKO, Aurora A MAb NCL-L-AK2 (clone JLM28) from Novocastra Laboratories (Newcastle, UK), Plk1 MAb (clone 35-206) and Histone H3 phosphorylated on Serine 10 (H3S10ph) polyclonal antibody from Upstate (Lake Placid, NY, USA).

### Cell culture and synchronisation

Human MCF-7 breast epithelial adenocarcinoma cells (HTB-22; ATCC, Teddington, UK) were cultured in EMEM (Gibco-BRL, Invitrogen, Carlsbad, CA, USA) supplemented with 2 mM glutamine, 1% non-essential amino acids, 10% FCS, 100 U ml^−1^ penicillin and 0.1 mg ml^−1^ streptomycin.

### Preparation of protein extracts and immunoblotting

MCF-7 cells were harvested by treatment with trypsin, washed in PBS and resuspended in lysis buffer (50 mM Tris-Cl, pH 7.5, 150 mM NaCl, 20 mM EDTA, 0.5% NP-40) at 2 × 10^7^ cells/ml. After incubation on ice for 30 min, the lysate was clarified by centrifugation (13 000 **g**, 15 min, 4°C). Lysates were separated by 4–20% SDS–PAGE (75 *μ*g protein/well) and immunoblotted as described (Stoeber *et al*, 2001). Blocking, antibody incubations and washing steps were performed using the following conditions: PBS/0.1% Tween-20/5% milk for Mcm2, Aurora A and Plk1; PBS/1% Tween-20/10% milk for geminin; and PBS/5% milk for H3S10ph.

### Immunohistochemistry

Archival formalin-fixed, paraffin-embedded tissue (PWET) obtained at initial diagnosis was available for all patients, and for each specimen, a block was chosen that contained a representative sample of invasive tumour. Consecutive serial sections cut from each PWET block were used for immunohistochemistry. Sections of 3-*μ*m thickness were cut onto Superfrost Plus slides (Visions Biosystems, Newcastle Upon Tyne, UK), dewaxed in xylene and rehydrated through graded alcohol to water. The tissue sections were pressure-cooked in 0.1 M citrate buffer at pH 6.0 for 2 min and immunostained using the Bond™ Polymer Refine Detection kit and Bond™-Max automated system (Vision Biosystems). Primary antibodies were applied at the following dilutions: Ki67 (1 : 300), Mcm2 (1 : 2000), geminin (1 : 600), ER (1 : 200), PR (1 : 200), Aurora A (1 : 70), Plk1 (1 : 1000) and H3S10ph (1 : 300). Her-2 immunostaining was performed using the DAKO HercepTest™ (DAKO), according to the manufacturer's instructions. Coverslips were applied with Pertex mounting medium (CellPath Ltd, Newtown, Powys, UK). Incubation without a primary antibody was used as a negative control and colonic epithelial sections as positive controls.

### Protein expression profile analysis

Protein expression analysis was performed by determining the labelling index (LI) of the markers in each tumour as described (Dudderidge *et al*, 2005; Shetty *et al*, 2005; Kulkarni *et al*, 2007). Slides were evaluated at low-power magnification ( × 100) to identify the regions of tumour with the highest intensity of staining. From these selected areas, 3–5 fields at × 400 magnification were captured with a charged-coupled-device camera and analysis software (SIS, Münster, Germany). Images were subsequently printed for quantitative analysis, which was performed with the observer unaware of clinicopathological variables. Both positive and negative cells within the field were counted and any stromal or inflammatory cells were excluded. Criteria for identification of positive cells were dependent on the biomarker: for Ki67, Mcm2, geminin and H3S10ph, cells with any degree of nuclear staining were scored positive; for Aurora A and Plk1, cells with any degree of nuclear or cytoplasmic staining were scored positive. A minimum total of 500 cells were counted for each case. The LI was calculated using the following formula: LI=number of positive cells/total number of cells × 100 as described (Kulkarni *et al*, 2007). To evaluate ER and PR expressions, the quick (Allred) score system was used and positivity was defined as a quick score of >3 as described earlier (Harvey *et al*, 1999; Gown, 2008). Her-2 protein overexpression was assessed using the Food and Drug Administration (FDA)-approved scoring system recommended by DAKO. The reassessment of 10 randomly selected cases by an independent assessor showed high levels of concordance.

### DNA image cytometry

For each case, one 40 *μ*m section of PWET, obtained from the same block as that assessed by immunohistochemistry, was used to prepare nuclei as described (Haroske *et al*, 1998). The Fairfield DNA Ploidy System (Fairfield Imaging Ltd, Nottingham, UK) was used for image processing, analysis and classification as described (Haroske *et al*, 1998). Lymphocytes and plasma cells were included as internal controls and 40 *μ*m sections of high-grade bladder tumour and normal colonic tissue as external controls for aneuploid and diploid populations, respectively. Histograms were classified according to the published criteria (Haroske *et al*, 1998) by two independent assessors with a high level of agreement without the knowledge of clinicopathological variables. For statistical analysis, tetraploid and polyploid tumours were grouped together with aneuploid tumours.

### Statistical analysis

Biomarker labelling indices were summarised with the median and interquartile range. The Mann–Whitney *U*-test was used to compare each marker with lymph node stage, ploidy status and with grade 3 against the normal sample. The Jonckheere–Terpstra non-parametric test for trend was used to compare markers across grade and Her-2 status. Spearman's rank correlation coefficient was used to assess associations between markers and NPI. The *χ*^2^-test for a linear-by-linear association with 1 d.f. was used to test for association between Her-2 and ploidy status. The unpaired *t*-test was used to compare mean NPI according to the ploidy status. Linear regression was used to assess for trend in mean NPI across Her-2. Cox regression was used in the analysis of disease-free survival and overall survival to provide hazard ratios and to assess the prediction of markers, split into two categories at the median, both in univariate models and in multivariate models adjusting for NPI. Kaplan–Meier plots were used to show the estimated predictive effects of markers ignoring, and also stratified by, the NPI category. Relationships between cell-cycle phenotype and clinical parameters were assessed using Pearson's *χ*^2^-test for grade, positive nodes and Her-2 status; using one-way analysis of variance for age, size and NPI; and using the Jonckheere–Terpstra test for ER and PR. All tests were two-sided, with effects summarised using 95% confidence intervals and assessed as statistically significant at the 5% level using SPSS software (version 15.0, SPSS Inc., Chicago, IL, USA).

## Results

### Cell-cycle-phase progression analysis of normal and malignant breast tissues

Monospecificity of antibodies against Mcm2, geminin, Plk1, Aurora A and the Aurora kinase substrate H3S10ph was confirmed in total cell extracts from asynchronous MCF-7 breast cancer cells by detection of a single protein with a molecular mass consistent with the reported electrophoretic mobility of the corresponding human antigen (Supplementary Figure 1A). Next, we studied the expression of these G1–S and G2–M regulators in normal breast specimens after reduction mammoplasty and in poorly differentiated, aggressive high-grade (grade 3) tumours. High levels of Mcm2 protein were detected in epithelial cells of the terminal duct lobular unit (TDLU), indicating that these cells reside in an in-cycle state (median: 33.5%) and not in G0 state (Stoeber *et al*, 2001; Blow and Hodgson, 2002; Gonzalez *et al*, 2005; Williams and Stoeber, 2007). However, whereas Mcm2 levels were high, Ki67 was expressed at low levels (median: 2.8%). It is of note that geminin, Aurora A, Plk1 (S–G2–M-phase makers) and H3S10ph (M-phase marker) were only expressed in a very small fraction of cells (<1%) of the TDLU, indicating a block to cell-cycle progression (Supplementary Figure 1B). This cell-cycle phenotype is in keeping with a G1-delayed/arrested state as reported earlier (Stoeber *et al*, 2001; Gonzalez *et al*, 2004; Shetty *et al*, 2005; Williams and Stoeber, 2007). A higher proportion of tumour cells expressed the Mcm2 licensing factor when compared with the normal mammary epithelium, indicating a greater number of cells engaged in a cell cycle (median values: Mcm2: 92.3 *vs* 33.5%, *P*<0.001). However, in contrast to normal breast tissue, high-level Mcm2 expression was also coupled to a high-level expression of the S–G2–M markers, geminin, Aurora A, Plk1, and the mitotic marker, H3S10ph, in breast cancer (median values: geminin (0.98 *vs* 17.4%, *P*<0.001), Aurora A (0 *vs* 11.7%, *P*<0.001), Plk1 (0.37 *vs* 14.2%, *P*<0.001) and H3S10ph (0 *vs* 2.5%, *P*<0.001)), a phenotype indicative of active cell-cycle progression (Wohlschlegel *et al*, 2002; Gonzalez *et al*, 2004; Wharton *et al*, 2004; Dudderidge *et al*, 2005; Obermann *et al*, 2005; Kulkarni *et al*, 2007; Williams and Stoeber, 2007) (Supplementary Figure 1B). Although Ki67 levels were low in the G1-arrested or -delayed state, characterising the normal mammary epithelium, high levels of Ki67 were associated with the actively cycling tumour phenotype (Ki67: 2.8 *vs* 40.2%, *P*<0.001).

### Relationship between cell-cycle state, tumour differentiation status, genomic instability and metastasis

#### Cell-cycle state and tumour differentiation

The clinicopathological characteristics of the study cohort are summarised in Supplementary Table 1, which includes grade and associated receptor status. First, we examined the relationship between expressions of the G1–S and G2–M regulators, and tumour grade. Expression levels of Mcm2, geminin, Plk1, Aurora A and H3S10ph were strongly associated with tumour grade (Supplementary Table 2). Arrested tumour differentiation was linked to the loss of ER and PR hormone receptor expressions and an increase in the expressions of G1–S and G2–M regulators. This indicates that increasing grade is coupled to an increase in the proportion of tumour cells engaged in the cell division cycle. This is in keeping with our observations in the HL60 differentiation model system that engagement of the somatic differentiation programme in tumour cells is coupled to G1 arrest and cell-cycle withdrawal into the out-of-cycle state, a process that is linked to repression of the DNA replication licensing machinery (Barkley *et al*, 2007). Interestingly, there was a significant overlap in the distribution of these cell-cycle proteins between grades (e.g., Aurora A and Plk1 levels, Supplementary Figure 2), which has important implications for exploitation of these molecules as predictors of therapeutic response to cell-cycle-phase-specific mechanistic agents (see Discussion). Expression levels of Ki67, Mcm2, geminin, Aurora A, Plk1 and H3S10ph showed a strong positive correlation, and those of ER and PR a negative correlation, with increasing NPI score, consistent with their link to differentiation status (Supplementary Table 3). Surprisingly, Her-2 expression did not show linkage to any of the G1–S or G2–M cell-cycle regulators, despite these factors acting downstream of mitogenic growth-signalling pathways, but there was a strong inverse association with PR expression (*P*<0.001) (Supplementary Table 4) and with increasing NPI score (Supplementary Table 5).

#### Cell-cycle state and genomic instability

A highly significant association between tumour grade and genomic instability (ploidy status) was observed (*P*<0.001). To investigate the relationship between G1–S and G2–M regulators and genomic instability, we linked their expression profiles to tumour DNA content (Supplementary Table 6). There was a highly significant association between the expression levels of all six cell-cycle biomarkers including Ki67 and aneuploidy (Ki67: *P*<0.001, Mcm2: *P*=0.009, geminin: *P*<0.001, Aurora A: *P*<0.001, Plk1: *P*=0.002 and H3S10ph: *P*<0.001). This indicates an increased engagement of tumour cells in the cell division cycle for malignancies exhibiting genomic instability, when compared with diploid tumours (Kulkarni *et al*, 2007; Williams and Stoeber, 2007). A weak association was observed between aneuploidy and increasing Her-2 expression (*χ*^2^=3.03, *P*=0.08), and between aneuploidy and increasing NPI score (Supplementary Table 5).

#### Cell-cycle state and loco-regional metastasis

No significant association was found between Ki67, Mcm2, geminin, Aurora A or Plk1 expressions and lymph node metastasis, but a weak association with the H3S10ph expression was observed (*P*=0.02) (Supplementary Table 7). There was a strong inverse association with ER (*P*=0.007) and PR (*P*=0.005) expressions and lymph node metastasis.

### Relationship between cell-cycle state, tumour DNA ploidy status and patient outcome

#### Univariate analysis

In our patient cohort, the NPI score was a strong predictor of disease-free survival and overall survival, with the hazard of relapse increasing just below two-fold per unit of NPI score (HR=1.81 (1.47–2.23), *P*<0.001), and the hazard of dying increasing just above two-fold per unit of NPI score (HR=2.15 (1.61–2.88), *P*<0.001) (Supplementary Table 8, Figure 2A). Patient's age was not a predictive factor. Ki67, Mcm2, geminin, Aurora A, Plk1 and H3S10ph expressions were identified as strong predictors of disease-free survival (HR=2.77 (1.44–5.30), *P*=0.002; HR=3.00 (1.56–5.76), *P*<0.001; HR=3.93 (1.98–7.80), *P*<0.001; HR=3.31 (1.67–6.57), *P*<0.001; HR=4.48 (2.21–9.09), *P*<0.001; and HR=3.49 (1.76–6.92), *P*<0.001, respectively) (Figures 2B and C and 3). The corresponding associations with overall survival were also appreciable, but generally smaller and not statistically significant, reflecting the smaller number of these events (Mcm2: HR=2.32 (0.99–5.43), *P*=0.05; geminin: HR=2.43 (1.04–5.68), *P*=0.04; Aurora A: HR=2.18 (0.93–5.12), *P*=0.07; Plk1: HR=3.46 (1.37–8.71), *P*=0.009; H3S10ph: HR=3.29 (1.31–8.30), *P*=0.01). A lower hazard of relapse was observed in the diploid group, but this was not significant (HR=0.62 (0.33–1.18), *P*=0.14). There was a significant increasing trend in the hazard of relapse and death through increasing categories of Her-2 expression (HR=1.44 (1.13–1.83), *P*=0.003 and HR=1.40 (1.02–1.94), *P*=0.04, respectively).

#### Predictive value of biomarkers over and above NPI

Multivariate analysis shows that the effects of these cell-cycle-linked biomarkers remain statistically significant and predictive of disease-free survival even after adjusting for NPI. Ki67, Mcm2, geminin, Aurora A, Plk1 and H3S10ph were identified as strong independent predictors of disease-free survival over and above NPI (HR=2.13 (1.08–4.23), *P*=0.03; HR=2.22 (1.12–4.41), *P*=0.02; HR=2.64 (1.27–5.49), *P*=0.01; HR=2.82 (1.37–5.80), *P*=0.005; HR=3.31 (1.57–6.97), *P*=0.002; and HR=2.07 (1.02–4.20), *P*=0.04, respectively) (Figures 2D and 3). No added value was achieved by including two or more of these markers. However, there was a tendency towards Aurora A being additionally predictive over Plk1 and NPI, with an adjusted HR of 1.95 (0.90–4.23), *P*=0.091.

### Relationship between cell-cycle phenotype, clinicopathological variables and patient outcome

We found that the individual cell-cycle-phase-specific biomarkers are powerful independent prognostic markers in breast cancer. This raises the question whether the cell-cycle kinetics or cell-cycle phenotype of a tumour might also have an impact on the pathobiology of this particular tumour type. We have shown earlier in our *in vitro* DNA replication assays that downregulation of the Mcm2–7 licensing factors, constituents of the DNA helicase, is a ubiquitous downstream mechanism by which the proliferative capacity of cells is lowered, as cells exit the cell division cycle into quiescent (G0), differentiated or senescent out-of-cycle states (Stoeber *et al*, 1998, 2001; Blow and Hodgson, 2002; Kingsbury *et al*, 2005; Barkley *et al*, 2007; Williams and Stoeber, 2007). To determine the cell-cycle phenotype, we selected a cut point of 30% for Mcm2 protein expression to define a group (Mcm2 <30%, phenotype I) in which the majority of tumour cells reside in an out-of-cycle state (Supplementary Figure 3, Figure 4). This group (phenotype I), 18% of all tumours, had geminin levels of <7%. This is in keeping with our observations in *in vitro* assays and self-renewing tissues that geminin is also tightly downregulated as cells enter quiescent (G0) and differentiated out-of-cycle states (Eward *et al*, 2004; Kingsbury *et al*, 2005; Barkley *et al*, 2007; Williams and Stoeber, 2007) (Figure 4, Table 1). In contrast, most cancers had Mcm2 expression levels above 30% (Mcm2 >30%) in which a majority of tumour cells reside in an in-cycle state (Williams and Stoeber, 2007) (Supplementary Figure 3, Figure 4, Table 1). Overall 58% of these tumours (phenotype III) displayed an active cell-cycle progression indicated by geminin levels above 7%, a cut point defined by the LI for the out-of-cycle state (Figure 4, Table 1). It is of note that a large number of breast cancers (phenotype II), 24% of all tumours, displayed an in-cycle phenotype (Mcm2 >30%) but expressing geminin levels below 7%, indicative of a G1-delayed or -arrested state (Stoeber *et al*, 2001; Blow and Hodgson, 2002; Gonzalez *et al*, 2004; Dudderidge *et al*, 2005; Shetty *et al*, 2005; Williams and Stoeber, 2007) (Figure 4, Table 1). Importantly, the distribution of the other S–G2–M biomarkers between the three groups exactly mirrors that observed for geminin, further reinforcing segregation into three distinct cell-cycle phenotypes (Figure 4).

Next, we investigated whether the cell-cycle phenotype influences *in vivo* behaviour and its association with clinicopathological variables including NPI. It is of note that there was no association with age, tumour size, lymph node metastasis, ER/PR or Her-2 receptor status. However, a greater proportion of grade 3 tumours, those exhibiting arrested differentiation, displayed the actively cycling phenotype. This cell-cycle profile was also associated with a higher NPI score (*P*<0.001) (Table 1). Univariate and multivariate analyses adjusted for NPI also indicated that the cell-cycle phenotype was a strong predictor of disease-free survival. The actively cycling phenotype (phenotype III) showed a much higher hazard of relapse than phenotypes I and II on both univariate and multivariate analyses (HR=3.90 (1.81–8.40), *P*<0.001 and HR=2.71 (1.81–6.23), *P*=0.019, respectively) (Figure 5). Intriguingly, an almost identical low hazard of relapse was observed between well-differentiated out-of-cycle tumours and high-grade tumours exhibiting a G1-delayed/arrested phenotype (phenotypes I and II) (HR=1.00 (0.22–4.46), *P*=0.99; Figure 5). The actively cycling phenotype was a superior predictor of recurrence to Ki67 on univariate analysis (HR of 3.90 vs 2.77) and remained so after adjusting for Ki67 following multivariate analysis (HR=2.98 (1.29–6.91), *P*=0.011), using the median value of 24% as a cut point. The actively cycling phenotype also remained a powerful predictor of recurrence on multivariate analysis even after adjusting for Ki67 at a 15% threshold (HR=2.80 (1.16–6.78), *P*=0.023), a lower cut point that some studies have identified as an optimal cutoff value for Ki67 prognostication (Ahlin *et al*, 2007; De Azambuja *et al*, 2007). On the contrary, multivariate analysis showed that Ki67 was not significantly predictive of disease recurrence over and above the actively cycling phenotype at either the 24 or 15% cut points (*P*=0.13 and *P*=0.19, respectively). It is of note that a strong and significant association was observed between breast cancer subtype and cell-cycle phenotype (*P*<0.001). The proportion of patients with an actively cycling phenotype (phenotype III) was significantly higher in both the Her-2 (91%, 10 out of 11) (*P*=0.003) and triple negative subtypes (96%, 25 out of 26) (*P*<0.001) than in the luminal subtype (49%, 71 out of 145) (Figure 6). Although the proportion of hormone receptor-negative tumours displaying the out-of-cycle phenotype (phenotype I) and the G1-delayed/arrested phenotype (phenotype II) was only 4% (1 out of 26) and 9% (1 out of 11), respectively, in the luminal subtype, the proportion was 51% (74 out of 145), of which 21% (30 out of 145) displayed phenotype I and 30% (44 out of 145) phenotype II (Figure 6).

## Discussion

Multiparameter analysis of core constituents of the cell-cycle machinery that regulate the G1–S and G2–M transitions provides a unique and detailed picture of the cell-cycle state of dynamic tumour cell populations *in vivo*. We selected breast cancer as a tumour model system in the first instance to test our novel cell-cycle algorithm because of the urgent demand for new prognostic and predictive markers in this tumour type. Given the rising cost of health care, as well as the recognition of both an individual's genetic variation and the significant biological heterogeneity of this disease, further improvements in adjuvant treatment will inevitably require individualised therapeutic decisions. The best prognostic tool described to date for identifying which patients are most likely to benefit from adjuvant chemotherapy is the NPI. More recent analysis of additional factors, such as oestrogen, progesterone and HER2/NEU receptor status, has only marginally improved prognostic assessment (Subramaniam and Isaacs, 2005), and data emerging from global gene expression profiling, although initially encouraging (Perou *et al*, 1999; Van ‘t Veer *et al*, 2002), have not provided a significantly improved prognostic assessment to date (Michiels *et al*, 2005, 2007; Dunkler *et al*, 2007). Therefore, there is an urgent requirement to identify new biomarkers, which can identify those patients at most risk of relapse and who are therefore most likely to benefit from toxic chemotherapeutic interventions.

Application of our novel cell-cycle algorithm to breast cancer has revealed major insights into the cell-cycle kinetics of this disease. Arrested differentiation and increasing genomic instability, hallmarks of more aggressive tumours, was associated with increasing levels of both G1–S and G2–M biomarker expressions, indicative of an increased engagement of tumour cells in the mitotic cell division cycle. These cell-cycle biomarkers were all identified as strong independent prognostic markers over and above the prognostic value of the NPI score alone. It is of note that the S–G2–M progression markers, Aurora A and Plk1, were identified as particularly powerful independent prognostic markers, in keeping with their higher expression levels in actively cycling tumour cell populations (Kulkarni *et al*, 2007; Williams and Stoeber, 2007). Importantly, our analysis revealed that breast cancer can be sub-grouped into three unique cell-cycle phenotypes, which impact on the pathobiology of these tumours. The actively cycling phenotype had a significantly higher risk of relapse when compared with out-of-cycle and G1-delayed/arrested phenotypes. The cell-cycle phenotype was also found to markedly outperform Ki67 as a predictor of recurrence in this tumour series. The cell-cycle phenotype was not associated with age, tumour size, loco-regional metastasis, ER/PR or Her-2 status. However, the actively cycling phenotype was linked to a higher NPI score because of the greater proportion of grade 3 tumours in this subgroup. The strong association between tumour grade and S–G2–M cell-cycle-phase progression mirrors the results of recent gene expression profiling studies applied to histological grade using a bottom-up supervised approach (Sotiriou *et al*, 2006; Sotiriou and Piccart, 2007). A gene-expression signature of 97 unique genes was identified allowing patients to be reclassified into two groups with high and low risk of recurrence. It is of note that grade 3 tumours displayed a gene expression profile significantly enriched with genes associated with cell-cycle progression and proliferation (Sotiriou *et al*, 2006).

There was a highly significant association between breast cancer subtype and cell-cycle phenotype. The aggressive cell-cycle phenotype III (actively cycling), strongly linked to a poor prognosis in our patient cohort, was much more highly represented in Her-2 subtype and triple negative tumours (hormone receptor-negative breast cancer) when compared with breast cancers of the luminal subtype. Deregulated cell-cycle control and active cell-cycle progression, therefore, appear to underlie the aggressive *in vivo* behaviour of these hormone receptor-negative poor prognostic subtypes. The cell-cycle phenotype, therefore, appears to represent a novel and unique independent parameter in breast cancer and is of major prognostic significance.

Interestingly, the normal mammary epithelium mimics the G1-delayed/arrested phenotype observed in breast cancer. Although the growth fraction identified by the standard proliferation marker Ki67 is small, a large number of mammary epithelial cells within the TDLU express Mcm2, indicating that a large number of cells appear to be replication licensed and therefore in-cycle (Stoeber *et al*, 2001; Blow and Hodgson, 2002; Shetty *et al*, 2005; Williams and Stoeber, 2007). However, these cells fail to express markers of cell-cycle progression, including the S–G2–M markers, geminin, Aurora A and Plk1, and the mitotic marker, H3S10ph, indicating that these cells reside in a G1-prolonged or -arrested state (Williams and Stoeber, 2007). We and others have suggested earlier that this primed, licensed state in non-proliferating breast may be an evolutionary adaptation allowing for a rapid response to pregnancy, with the consequence that failure to downregulate the DNA replication licensing pathway may increase the risk of transition to uncontrolled cellular proliferation (Stoeber *et al*, 2001; Blow and Hodgson, 2002). It is noteworthy in this context that a recent study exploiting a hypomorphic viable allele of Mcm4 was found to cause chromosomal instability and mammary adenocarcinomas in mice (Shima *et al*, 2007).

**Figure 1:**
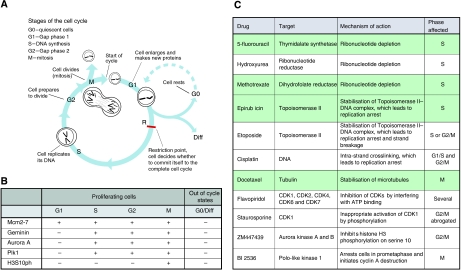
(**A**) Diagrammatic representation of the mitotic cell division cycle. (**B**) Phase-specific distribution of cell-cycle biomarkers in proliferating cells and out-of-cycle states. (**C**) Cell-cycle-phase-specific chemotherapeutic and mechanistic agents. Drugs highlighted in green are commonly used in the treatment of breast cancer.

**Figure 2:**
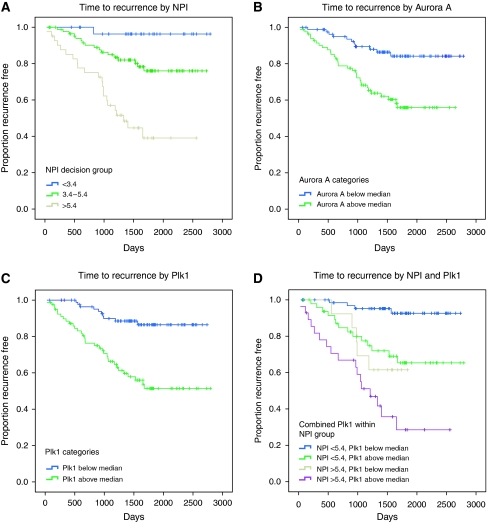
Kaplan–Meier curves showing association between NPI, Plk1 and Aurora A and disease-free survival (days from diagnosis to death, recurrence or last follow-up) across the whole series. (**A**) NPI and disease-free survival segregated into the three decision group categories, *P*<0.001. (**B**) Aurora A and disease-free survival stratified by those patients lying above or below the median LI; HR=3.31 (1.67–6.57), *P*<0.001. (**C**) Plk1 and disease-free survival stratified by those patients lying above or below the median LI; HR=4.48 (2.21–9.09), *P*<0.001. (**D**) Effect of Plk1 after adjustment for NPI; HR=3.31 (1.57–6.97) risk above Plk1 median relative to below median, *P*=0.002.

**Figure 3:**
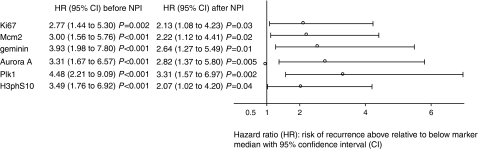
Effect of cell-cycle biomarkers on disease-free survival before and after adjusting for NPI. All biomarkers are associated with disease-free survival. The effects remain statistically significant after adjusting for NPI, so that they are predictive, independent of NPI.

**Figure 4:**
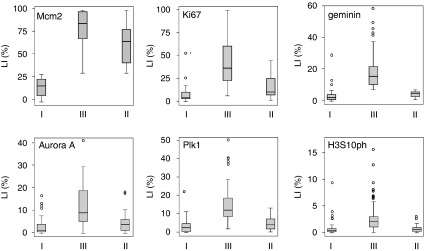
Distribution of cell-cycle biomarker expression defines three distinct cell-cycle phenotypes: (I) out-of-cycle state, (II) in cycle G1-delayed/arrested state and (III) actively cycling state. The median (solid black line), interquartile range (boxed) and robust range excluding outlying cases (enclosed by lines) are shown. Outlying cases are shown by isolated points (LI, labelling index).

**Figure 5:**
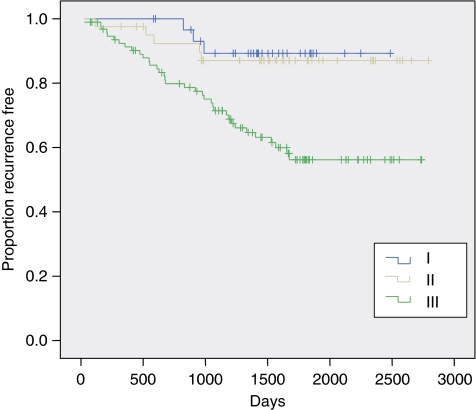
Kaplan–Meier curves showing association between cell-cycle phenotype and disease-free survival. (I) Out-of-cycle state, (II) in cycle G1-delayed/arrested state and (III) actively cycling state. On univariate analysis, comparing phenotype III with phenotypes I and II combined, HR=3.90 (1.81–8.40), *P*<0.001. On multivariate analysis, adjusted for NPI, HR=2.71 (1.18–6.23), *P*=0.019.

**Figure 6:**
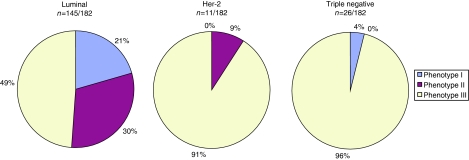
Relationship between cell-cycle phenotype and breast cancer subtypes. The panels show the proportion of each breast cancer subtype, which display cell-cycle phenotypes I (out of cycle), II (G1-delayed/arrested) and III (actively cycling). It is of note that the majority of Her-2 and triple negative tumours display the actively cycling phenotype (III).

**Figure 7:**
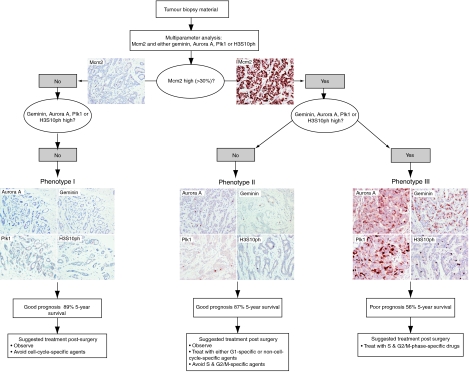
Proposed prognostic and predictive cell-cycle-phase algorithm in breast cancer. Three distinct cell-cycle phenotypes characterised by the differential expression of cell-cycle biomarkers Mcm2, Aurora A, geminin, Plk1 and H3S10ph. Prognosis and treatment response can be predicted from the distinct immunoexpression profile displayed by each tumour.

In addition to the strong prognostic significance of our biomarker algorithm, cell-cycle profiling of tumours has potential as a predictor of therapeutic response to cell-cycle-phase-specific agents (Figure 7). It is becoming increasingly apparent from disappointing intent-to-treat analyses of large conventionally designed trials, such as TACT (Ellis *et al*, 2007) and tAnGo (Poole *et al*, 2008) that further improvements in adjuvant treatment will inevitably require individualised therapeutic decisions. If there is a test to predict resistance to a drug or to target the patient selection for a clinical trial, the risk of clinical failure declines considerably. On the basis of our study, we hypothesise that the panel of cell-cycle-regulated biomarkers discussed here may allow the prediction of treatment response to cell-cycle-phase-specific chemotherapeutic agents, including small-molecule inhibitors targeting the cell-cycle machinery (Figure 1C). It appears from our data that a proportion of patients with high-grade breast cancer receiving adjuvant chemotherapy may not gain significant benefit from present drug regimes. An almost identical low hazard of relapse was observed between patients with well-differentiated out-of-cycle tumours who are often spared chemotherapy and those with high-grade tumours exhibiting a G1-delayed/arrested phenotype. Patients with high-grade tumours, but falling into the G1-delayed/arrested phenotype group, representing 24% of patients, are perhaps less likely to benefit from S and M cell-cycle-phase-targeted agents and can, therefore, be spared these toxic therapies. These patients are more likely to benefit from G1-phase-targeted agents or non-cell-cycle-specific anticancer drugs. Moreover, it is only patients showing the actively cycling, aggressive cell-cycle phenotype, and that are transiting S–G2–M phase, those are likely to benefit from conventional S- or M-phase-directed agents or from the new generation of Aurora and Plk1 mitotic kinase inhibitors that are now entering clinical trials (Keen and Taylor, 2004; Plyte and Musacchio, 2007). This is in keeping with our observations that the aggressive cell-cycle phenotype III (actively cycling) was strongly associated with Her-2 and triple negative subtypes, and it is these tumours that are characterised by high neoadjuvant response rates (Carey *et al*, 2007). Interestingly, in our patient cohort, 29% of patients with phenotype I, 20% of patients with phenotype II and 54% of patients with phenotype III tumours received cell-cycle-phase-specific agents in the adjuvant setting. If our prediction about cell-cycle phenotype and therapeutic benefit is correct, this would suggest that 18 out of 76 patients (phenotypes I and II) have received adjuvant therapy for little gain and that 49 out of 106 patients (phenotype III) may have been undertreated, that is, would have benefited from cell-cycle-phase-specific agents. Presently, there is persistent controversy about whether adjuvant chemotherapy benefits patients bearing ER-positive tumours or not (Anonymous, 2005, 2007). Interestingly, in our analysis, 49% of the hormone receptor-positive luminal subtype exhibited the actively cycling phenotype (phenotype III) (Figure 6), suggesting that many of these patients would derive benefit from cell-cycle-phase-specific chemotherapeutic agents.

Our cell-cycle-phase analysis suggests that a simple biomarker algorithm can be applied to breast cancer to improve prognostic assessment over and above NPI, and to help identify those patients who are most likely to benefit from adjuvant chemotherapy (Figure 7). Importantly, cell-cycle phenotyping is readily applicable to routinely processed surgical biopsy material in the hospital histopathology laboratory and is amenable to high-throughput screening, using automated immunostaining platforms and quantitative image analysis. This has distinct advantages over genomic array profiling, which requires specialised laboratory facilities for RNA and/or DNA preparation, microarray platforms and bioinformatics support. Moreover, fresh material is required for RNA/DNA analysis, which disrupts the surgical specimen, potentially compromising morphological diagnosis, assessment of surgical margins of excision and staging. This is a particular problem for small samples such as those obtained using core needle biopsy techniques.

In conclusion, the biomarker algorithm discussed here provides novel insights into the cell cycle state of dynamic tumour cell populations *in vivo*. This algorithm has enabled us to identify three unique cell-cycle phenotypes in breast cancer, a new and independent parameter in this tumour type, which is of major prognostic significance and outperforms Ki67. In particular, deregulated cell-cycle control and accelerated cell-cycle progression appears to underlie the aggressive *in vivo* behaviour of the hormone receptor-negative Her-2 and triple negative subtypes (Sorlie *et al*, 2003; Carey *et al*, 2007). Further studies are now in progress to test the predictive value of this algorithm, assessing response to cell-cycle-phase-specific agents in both the adjuvant and neoadjuvant settings.

## Figures and Tables

**Table 1:** Relationship between cell-cycle phenotype and clinicopathological parameters

	**I (out-of-cycle)**	**II (in-cycle G1-delayed/arrested)**	**III (actively cycling)**
	**Mcm2 <30%**	**Mcm2 ⩾30%**	**Mcm2 ⩾30%**
		**Geminin <7%**	**Geminin ⩾7%**
	***N*=33 (18%)**	***N*=44 (24%)**	***N*=105 (58%)**
Age, mean (s.d.) (*P*=0.13)	61.9 (12.4)	61.2 (14.1)	57.4 (13.9)
			
*Grade (*P<0.001)^*^			
1	27% (9/33)	23% (10/44)	5% (5/105)
2	61% (20/33)	64% (28/44)	30% (32/105)
3	12% (4/33)	14% (6/44)	65% (68/105)
			
Size, mean (s.d.) (*P*=0.55)	24.7 (17.5)	29.1 (19.8)	28.0 (17.4)
			
Positive nodes (*P*=0.23)	39% (12/31)	33% (13/40)	48% (47/98)
			
NPI, mean (s.d.) (*P*<0.001)	3.8 (1.3)	4.0 (1.2)	4.9 (1.2)
			
ER+ cases^a^ (*P*=0.08)	100% (73.9–100%)	100% (100–100%)	88.9% (0–100%)
PR+ cases^a^ (*P*=0.14)	72.4% (35.8–100%)	92.2% (47–100%)	18.8% (0–97.8%)
			
*Her-2*			
0	66.7% (22/33)	68.2% (30/44)	53.3% (56/105)
1+	15.2% (5/33)	18% (8/44)	19% (20/105)
2+	9.1% (3/33)	4.5% (2/44)	7.6% (8/105)
3+ (*P*=0.45)	9.1% (3/33)	9.1% (4/44)	20% (21/105)

ER=oestrogen receptor; NPI=Nottingham Prognostic Index; PR=progesterone receptor.

^a^Median (interquartile ranges).

^*^Significant association restricted to phenotype III.
