# Supplementary figures and images for: Cell-cycle-phase progression analysis identifies unique phenotypes of major prognostic and predictive significance in breast cancer
Source: Br J Cancer. 2009 Feb 24;100(6):959–70. doi: 10.1038/sj.bjc.6604924 (PMC2661794; doi:10.1038/sj.bjc.6604924)

Supplementary Figure 1

**A**

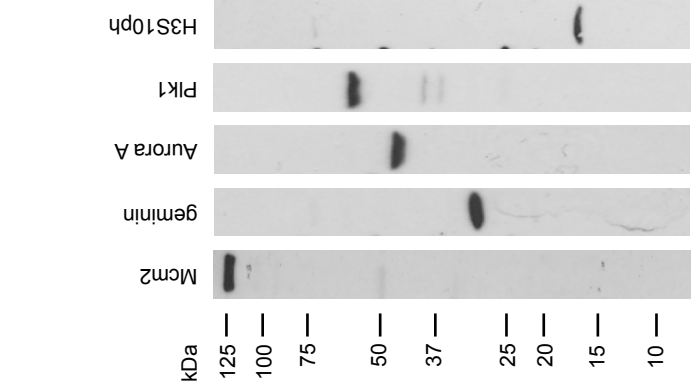

**B**

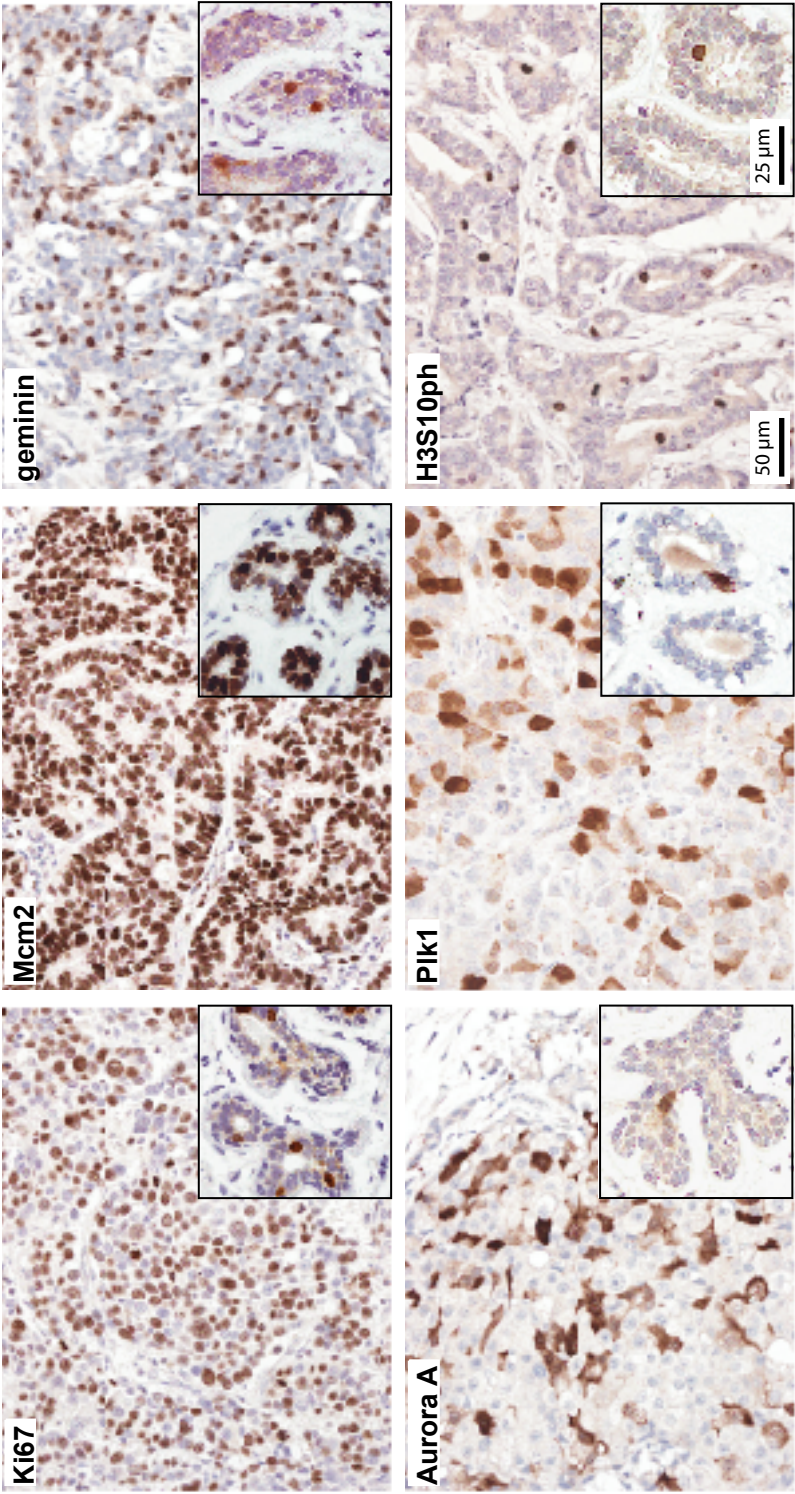

Supplement: Supplementary Figure 1 [file 6604924x1.pdf]

Supplementary Figure 2

A

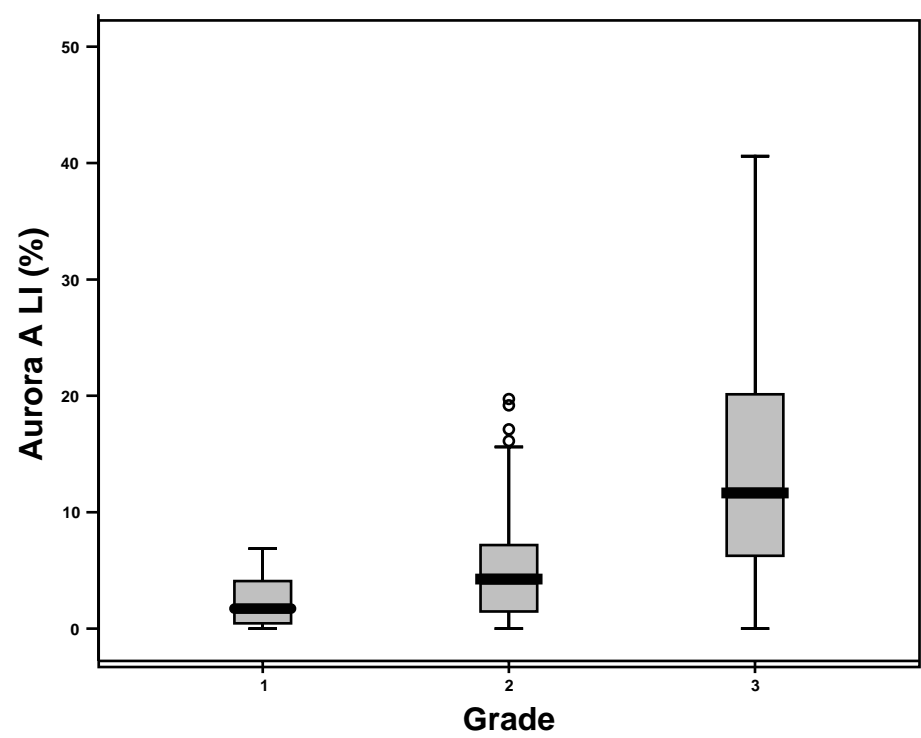

B

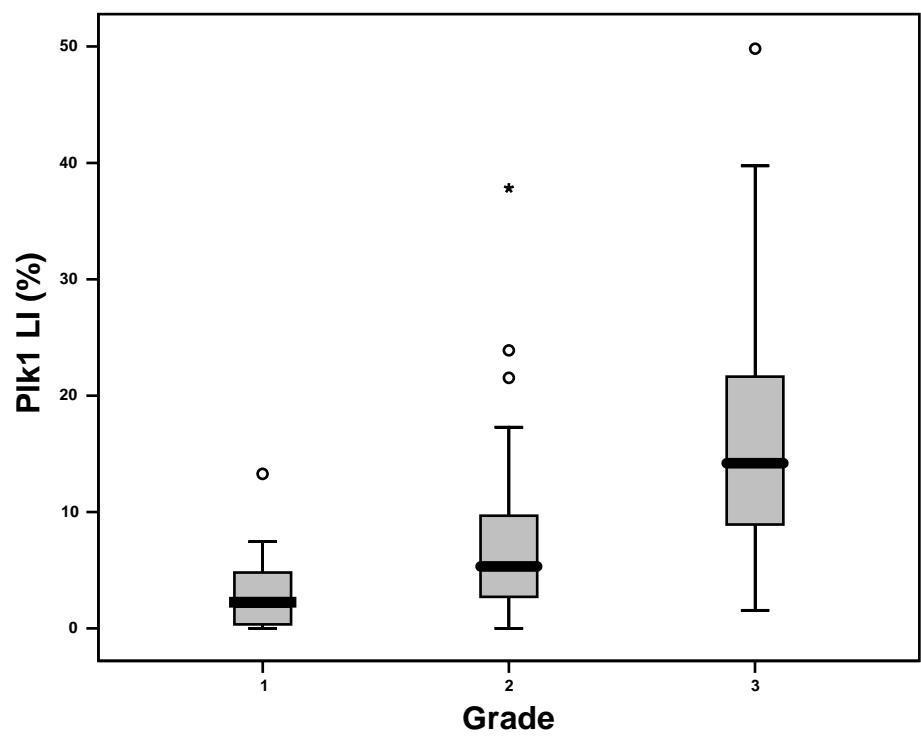

Supplement: Supplementary Figure 2 [file 6604924x2.pdf]

**Supplementary Figure 3**

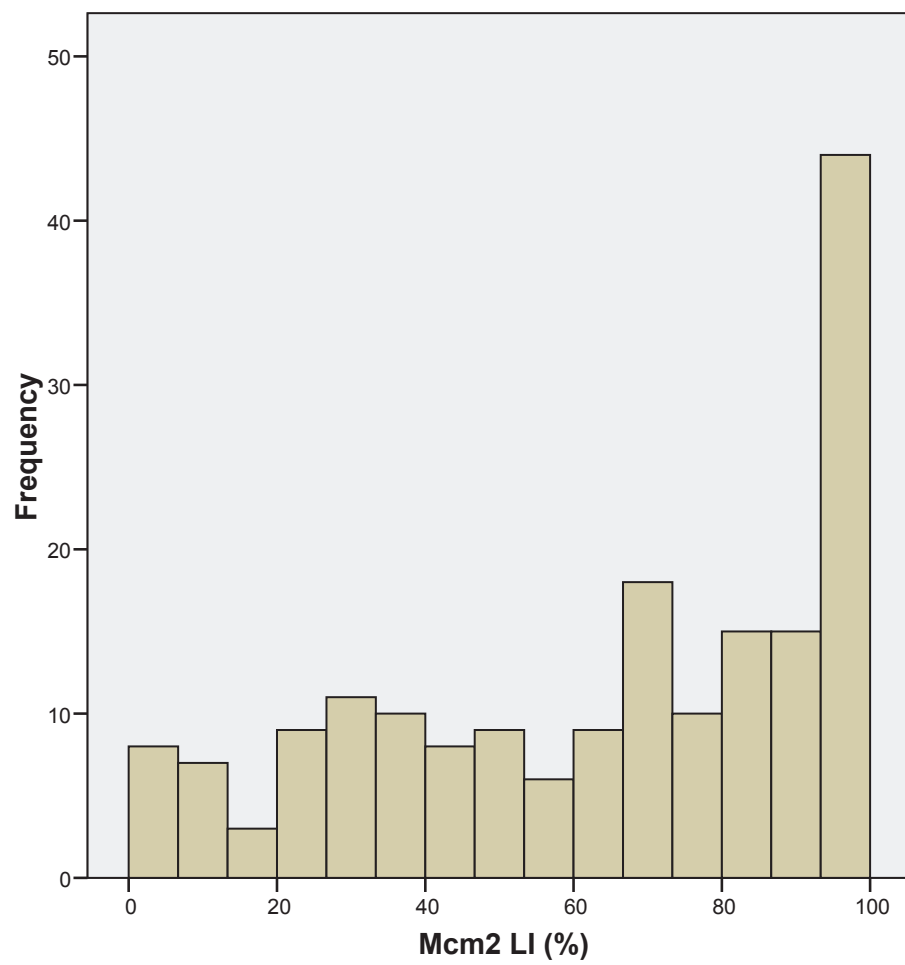

Supplement: Supplementary Figure 3 [file 6604924x3.pdf]
