# Supplementary material for: Cell-cycle-phase progression analysis identifies unique phenotypes of major prognostic and predictive significance in breast cancer
Source: Br J Cancer. 2009 Feb 24;100(6):959–70. doi: 10.1038/sj.bjc.6604924 (PMC2661794; doi:10.1038/sj.bjc.6604924)
Supplement: Supplementary Figures Legends [file 6604924x4.doc]

**SUPPLEMENTARY FIGURE LEGENDS**

**Supplementary Figure 1**: (**A**) Immunoblots of total cell lysates prepared from asynchronously proliferating MCF-7 with antibodies to Mcm2, geminin, Aurora A, Plk1 and H3S10ph. (**B**) Photomicrographs of paraffin-embedded tissue sections of grade 3 breast cancer immunohistochemically stained with antibodies to Ki67, Mcm2, geminin, Aurora A, Plk1 and H3S10ph (original magnification x 400). Insets show immunostaining of normal breast (magnification x 800).

**Supplementary Figure 2: Aurora A and Plk1 expression across tumour grades.** The median (solid black line), interquartile range (boxed), and robust range excluding outlying cases (enclosed by lines) of Aurora A and Plk1 expression are shown according to tumour grade (outlying cases are shown by isolated points (A and B).

**Supplementary Figure 3:**  **Distribution of Mcm2 expression in the study sample.** Frequency of Mcm2 protein expression across the breast cancer patient cohort. (Mean =64.4101, Std. Dev. = 30.4632, N =182).
