# Supplementary material for: Cell-cycle-phase progression analysis identifies unique phenotypes of major prognostic and predictive significance in breast cancer
Source: Br J Cancer. 2009 Feb 24;100(6):959–70. doi: 10.1038/sj.bjc.6604924 (PMC2661794; doi:10.1038/sj.bjc.6604924)
Supplement: Supplementary Tables 1–8 [file 6604924x5.doc]

**Supplementary Table 1: Patient characteristics**

|  |  | Frequency (number) |
| --- | --- | --- |
| Age (years)  Size (mm)  Lymph node stage  Grade  NPI score  Ploidy status  ER  PR  Her-2  Lymphovascular invasion  Tumour type  Breast cancer recurrence  Breast cancer death  Recurrence follow-up (years)  Survival follow-up (years) | <40  40-49  50-59  60-69  70+  <11  11-20  21-30  31-40  >40  Negative nodes  Positive, 1 to 3  Positive, 4 or more  Unknown  1  2  3  mean (sd): 4.49 (1.31)  <3.4  3.4-5.4  >5.4  Unknown  Diploid  Aneuploid  Unknown  Positive  Negative  Positive  Negative  0  1+  2+  3+  Absent  Present  Unknown  Invasive ductal  Lobular  Mucinous  Mixed  Micropap  Yes  No  Unknown  Yes  No  Unknown | 7% (12)  19% (34)  28% (51)  22% (41)  24% (44)  9% (16)  33% (60)  30% (54)  16% (29)  13% (23)  53% (97)  22% (40)  18% (32)  7% (13)  13% (24)  44% (80)  43% (78)  18% (32)  51% (93)  24% (44)  7% (13)  47% (86)  50% (90)  3% (6)  79% (144)  29% (38)  64% (117)  36% (65)  59% (108)  18% (33)  7% (13)  15% (28)  47% (86)  38% (69)  15% (27)  78% (142)  14% (26)  2% (4)  5% (9)  1% (1)  26% (48)  68% (124)  6% (10)  14% (26)  80% (146)  6% (10)  632 (3.8 per subject)  684 (4.1 per subject) |

**Supplementary** **Table 2: Relationship between biomarker expression* and tumour differentiation**

|  | Grade 1  (n=24) | Grade 2  (n=80) | Grade 3  (n=78) | p-value^†^ |
| --- | --- | --- | --- | --- |
| Ki67  Mcm2  Geminin  ER  PR  Aurora A  Plk1  H3S10ph | 7.3 (2.4-17.6) ^‡^  45.8 (17.1-65.6)  2.0 (0.9-5.8)  100.0 (100.0-100.0)  93.5 (52.8-100.0)  1.7 (0.4-4.1)  2.3 (0.3-5)  0.2 (0.03-0.5) | 16.6 (8.05-30.9)  57.8 (29.3-83)  6.5 (3.3-10.6)  100.0 (94.0-100.0)  87.9 (38.0-100.0)  4.3 (1.4-7.2)  5.3 (2.6-9.7)  0.8 (0.3-1.6) | 40.2 (25-66.9)  92.3 (70.0-100.0)  17.4 (10.6-24.8)  30.2 (0.0-100.0)  0.0 (0.0-90.3)  11.7 (6.2-20.2)  14.2 (8.9-21.7)  2.5 (1.4-3.8) | <0.001  <0.001  <0.001  <0.001  <0.001  <0.001  <0.001  <0.001 |

^†^Jonckheere-Terpstra test

*Labelling index (expressed as percentages)

^‡^Median (inter-quartile range)

**Supplementary** **Table 3: Relationship between biomarker expression and NPI**

|  | Expression value* defining the marker’s median | Mean NPI below marker’s median | Mean NPI above marker’s median | Correlation between marker and NPI^†^ |
| --- | --- | --- | --- | --- |
| Ki67  Mcm2  Geminin  ER  PR  Aurora A  Plk1  H3S10ph | 24.0  70.7  9.0  99.9  63.0  6.10  8.40  1.25 | 4.13  4.10  4.00  4.97  4.78  4.09  3.97  3.95 | 4.86  4.88  4.99  4.05  4.19  4.87  5.01  5.01 | +0.42, p<0.001  +0.40, p<0.001  +0.44, p<0.001  -0.39, p<0.001  -0.34, p=0.005  +0.39, p<0.001  +0.46, p<0.001  +0.47, p<0.001 |

*Labelling index (expressed as percentages)

^†^ non-parametric Spearman correlation coefficient

**Supplementary Table 4:** **Relationship between biomarker expression* and Her-2 status**

|  | 0  (n=108) | 1+  (n=33) | 2+  (n=13) | 3+  (n=28) | p-value^†^ |
| --- | --- | --- | --- | --- | --- |
| Ki67  Mcm2  Geminin  ER  PR  Aurora A  Plk1  H3S10ph | 21.4 (9.5-43.9)^‡^  70.5 (35.0-94.2)  8.4 (3.3-16.9)  100.0 (15.2-100.0)  81.2 (0.0-100.0)  5.8 (2.0-12.3)  7.5 (3.3-13.8)  0.9 (0.34-2.67) | 22.0 (7.2-35.8)  63.3 (38.9-80.1)  7.9 (4.0-13.3)  100.0 (71.0-100.0)  58.4 (0.4-95.1)  6.0 (1.0-11.8)  6.1 (3.4-12.5)  1.0 (0.46-2.60) | 24.6 (10.2-52.1)  75.0 (28.7-90.0)  10.6 (4.7-17.9)  96.4 (61.6-100.0)  45.8 (15.0-97.4)  5.9 (1.1-9.6)  10.2 (2.8-13.4)  1.7 (0.72-2.39) | 32.2 (19.7-48.2)  78.3 (54.8-95.6)  15.7 (7.8-21.7)  70.0 (0.0-100.0)  0.0 (0.0-52.6)  8.9 (4.5-13.1)  11.2 (8.8-17.0)  1.9 (0.98-2.82) | 0.17  0.67  0.056  0.12  <0.001  0.43  0.073  0.044 |

^†^Jonckheere-Terpstra Test

*Labelling index (expressed as percentages)

^‡^Median (inter-quartile range)

**Supplementary Table 5: Relationship of Her-2 status and ploidy status with NPI**

|  | Mean NPI | Difference in NPI means (95% CI), p-value |
| --- | --- | --- |
| Ploidy  Aneuploid (n=83)  Diploid (n=81) | 4.67  4.33 | Difference = 0.33 (-0.07 to +0.74), p=0.11* |
| Her-2  0 (n=101)  1+ (n=31)  2+ (n=11)  3+ (n=26) | 4.35  4.44  4.53  5.09 | Mean NPI is higher per category of Her-2 by  0.22 (0.04 to 0.40), p=0.014^†^ |

* Unpaired t-test

^†^ Linear regression test for linear trend

**Supplementary Table 6: Relationship between biomarker expression* and tumour DNA ploidy status**

| Biomarker | Aneuploid  (n=90) | Diploid  (n=86) | p-value^†^ |
| --- | --- | --- | --- |
| Ki67  Mcm2  Geminin  ER  PR  Aurora A  Plk1  H3S10ph | 30.0 (16.8-53.1) ^‡^  75.8 (48.8-96.7)  11.5 (5.9-21.9)  96.9 (8.0-100.0)  59.1 (0.0-97.0)  7.3 (3.5-14.9)  9.8 (4.8-15.7)  1.78 (0.78-2.89) | 17.5 (7.2-33.1)  63.1 (31.2-84.4)  6.9 (2.4-12.2)  100.0 (65.7-100.0)  69.3 (0.0-100.0)  4.1 (1.0-8.4)  7.1 (2.1-11.1)  0.70 (0.22-1.7) | <0.001  0.009  <0.001  0.054  0.12  <0.001  0.002  <0.001 |

^†^Mann-Whitney Test

*Labelling index (expressed as percentages)

^‡^Median (inter-quartile range)

**Supplementary Table 7: Relationship between biomarker expression* and**

**lymph node stage**

|  | Negative  (n=97) | Positive  (n=72) | p-value^†^ |
| --- | --- | --- | --- |
| Ki67  Mcm2  Geminin  ER  PR  Aurora A  Plk1  H3S10ph | 22.7 (9.5-43.3) ^‡^  70.3 (36.2-90.1)  7.9 (3.6-16.3)  100.0 (60.6-100.0)  74.3 (4.5-100.0)  5.3 (1.5-10.8)  7.5 (3.3-14.3)  0.94 (0.37-2.38) | 24.8 (11.1-47.3)  71.9 (42.2-98)  10.4 (5.1-18.2)  90.7 (0.0-100.0)  31.3 (0.0-96.1)  7.0 (2.9-14.0)  9.2 (4.2-14.7)  1.67 (0.68-2.93) | 0.49  0.25  0.22  0.007  0.005  0.16  0.22  0.02 |

^†^Mann-Whitney Test

*Labelling index (expressed as percentages)

^‡^Median (inter-quartile range)

**Supplementary Table 8: Effect of NPI on disease-free survival and overall survival**

|  | Relapse rate | Cancer death rate |
| --- | --- | --- |
| NPI  <3.4  3.4-5.4  >5.4 | 3% (1/30)  20% (17/87)  54% (22/41) | 3% (1/30)  7% (6/87)  34% (14/41) |
| HR*  (95% CI) | 1.79 (1.46 to 2.20), p<0.001 | 2.18 (1.63 to 2.93), p<0.001 |
| HR* after adjusting for age | 1.81 (1.47 to 2.23), p<0.001 | 2.15 (1.61 to 2.88), p<0.001 |

* per unit of NPI
